# Enhanced viscoelastic property of iron oxide nanoparticle decorated organoclay fluid under magnetic field

**DOI:** 10.1186/s40580-017-0116-z

**Published:** 2017-08-23

**Authors:** You-Hwan Son, Youngsoo Jung, Heesuk Roh, Jung-Kun Lee

**Affiliations:** 1 Department of Mechanical Engineering & Material Science, University of Pittsburgh, Pittsburgh, PA 15261 USA; 2 Present Address: Samsung Advanced Institute of Technology, Suwon, 443-803 Korea; 3 Present Address: OCI, Seoul, 100-718 Korea

## Abstract

Stable hydrophobic nanocomposites of magnetic nanoparticles and clay are prepared by the self-assembly of magnetite (Fe_3_O_4_) nanoparticles on surfaces of exfoliated clay platelets. Due to the attractive interaction between hydrophobic groups, oleic acid coated nanoparticles are strongly attached to the surface of cetyl trimethylammonium cation coated clay platelets in organic media. Crystal structure and magnetic property of composite particles are examined using electron microscopy, x-ray diffractometer and vibration sample magnetometer. In addition, composite particles are dispersed in mineral oil and rheological properties of composite particle suspensions are characterized using steady-state and oscillatory measurements. Magnetite nanoparticle decorated organoclay forms a tunable network in mineral oil. When a magnetic field is applied, the composite particle fluid exhibits higher storage modulus and maintains a solid-like property at larger strain. Our results show that the viscoelastic property of the magnetite nanoparticle decorated organoclay fluid is controlled by applying external magnetic field.

## Introduction

Nanophase magnetic materials have been extensively studied because of their potential applications [1–3]. Suspensions of the magnetic nanoparticles, so called ferrofluids, are regarded as smart materials, meaning that they can be rapidly and reversibly transformed between a fluid-like to a solid-like state within milliseconds by applying a magnetic field. Consequently, the suspensions of the magnetic nanoparticles show dramatic and tunable changes in rheological properties under the influence of the external applied magnetic field. In addition, functionalized γ-Fe_2_O_3_ nanoparticles have been studied for use in separating target materials magnetically, which is a more selective and efficient method than others such as centrifugation or filtration [4–7]. However, very fine iron oxide nanoparticles do not have large magnetic moment and the magnetic force of fine nanoparticles is not large enough to overcome Brownian motion in the fluid. At the same time, while large magnetic particles have large magnetic moment, they have a larger coercive field and remnant magnetization. Therefore, in larger particles, the relative change in the magnetization over an external magnetic field is small and the magnetic response is less sensitive to an external field than that of fine nanoparticles.

In this regard, magnetic nanoparticles arrays are attraction a huge amount of research attention from academia and industry. This is because the collective response of magnetic nanoparticles generates both large magnetic moment and high sensitivity to an external field, which is needed for many applications of magnetic materials. Several supporting materials have been studied to induce the assembly of the nanoparticles [8–12]. Clay minerals provide one of the best matrix materials on which nanoparticles can be collected and aligned in that clay minerals are abundant, environmentally friendly and economical [13–18]. Since plate type clay materials have different electric charges locally in water, the surface, edge or interlayer space of clay materials can be decorated with charged nanoparticles. When the magnetic nanoparticles were attached to clay minerals, a movement of nanoparticle-clay mixture was controlled by applying a magnetic field. Because of a tunable motion of the mixture and an absorbent ability of the clay, the mixture of magnetic nanoparticles and clay minerals was successfully used to separate contaminants in water [5, 7, 19].

To date, however, studies on the magnetic nanoparticle decorated clay have been mainly carried out in an aqueous fluid system. In organic solutions, the electrostatic interaction between clay and oxide nanoparticles is not strong and the viscosity of the base fluid is large. Consequently, it is difficult to stimulate a strong magnetic response by applying a small magnetic field to organic suspensions of a clay–magnetic nanoparticle mixture. In this study, we investigate the self-assembly of magnetite (Fe_3_O_4_) nanoparticles on the surface of hydrophobic clay in organic liquid, which does not require electrostatic interactions between the nanoparticles and the clay minerals. We also examine the magnetorheological behavior of the organic fluid of nanoparticle decorated hydrophobic clay (called organoclay). When plate-type montmorillonite and magnetite nanoparticles are treated with alkyl amine and oleic acid, magnetite nanoparticles are strongly attached to the surface of montmorillonite due to hydrophobic attraction. Such magnetite nanoparticle decorated clay is well dispersed in organic media and the rheological properties of the fluid are easily controlled by applying a magnetic field.

## Theoretical background on rheological properties of fluid systems

### Fluidic behaviors of clay suspensions—steady state shear measurement

Fluidic behaviors of suspensions are classified by a relationship between shear stress τ and shear rate $\dot{\gamma }$. The shear stress is stated precisely as the tangential force applied per unit area, and the shear rate is as the change of shear strain per unit time. The ratio of shear stress τ to shear rate $\dot{\gamma }$ is defined as viscosity *η*. In other words, η is a measure of the resistance of suspensions to shearing flow or fluidic motion.

1 $$\eta = \frac{\tau }{{\dot{\gamma }}}.$$

**Fig. 1:**
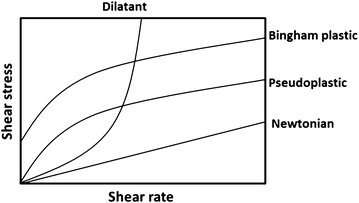
Consistency curves for four different types of flow systems

Figure 1 schematically shows five types of fluidic behaviors: Newtonian, pseudoplastic, Bingham plastic, Bingham, and Dilatant. In the Newtonian fluid, shear viscous stress is linearly proportional to shear rate and viscosity (i.e. a ratio of shear stress over shear strain) does not depend on the shear rate. Other four types of fluids are called non-Newtonian fluids which do not exhibit a constant shear stress–shear strain ratio. In many cases, aqueous clay suspensions exhibit Bingham plastic behavior [20].

Bingham model postulates that a finite stress should be applied to initiate flow. As the shear rate increases, Bingham fluid gets close to Newtonian fluid. Hence, the resistance of Bingham fluid to the shear flow can be set as two regimes; (i) a non-Newtonian regime in which the shear stress is constant regardless of the shear rate, (ii) a Newtonian regime in which the shear stress is proportional to the shear rate. The Bingham model is quantitatively expressed as follow,

2 $$\uptau = \uptau_{\text{B}} + \upeta_{pl} \times \dot{\gamma }.$$

where η_*pl*_ is the plastic viscosity that is determined by the slope of the curve, and τ_B_ is the Bingham yield stress estimated from the intercept of the flow curve at high shear rate regime. The other models describing the rheological behavior of clay suspensions are Casson equation [21].

3 $$\uptau^{ 1/ 2} = {\text{ k}}_{0} + {\text{ k}}_{ 1} \; \times \;\dot{\gamma }^{ 1/ 2} .$$

and the Herschel–Bulkley equation [22].

4 $$\uptau = \uptau_{\text{y}} + K \times \dot{\gamma }^{\text{n}}$$

where yield stress τ_y_, flow consistency *K*, and flow behavior indices n. Both models have been used to describe the consistency curves of the clay based fluids [23]. In both cases, the given suspension has an initial yield stress at low shear rates. As the shear rate increases, the viscosity of the fluid decreases and gets saturated to a certain value. This is called a shear thinning behavior.

### Characterization of viscoelastic behavior by oscillatory shear measurement

The non-Newtonian behavior of clay suspensions is due to rearrangement of clay particles in fluids. An effective way to examine this interparticle interaction in clay suspensions is to characterize viscoelastic properties of fluids by oscillatory shear measurement [24]. This method provides quantitative information on mechanical properties of soft materials such as colloidal suspension, gel, emulsion polymer and foam. The oscillatory measurement produces sinusoidal deformation in soft materials and measures applied stress and strain of soft materials. In pure elastic materials, stress is exactly in phase with a sinusoidal change in strain over time and a proportionality constant (shear modulus) does not depend on the magnitude of stress or strain rate. If materials show an ideal viscous behavior, stress for the deformation is proportional to a rate of deformation which is called a strain rate ($\dot{\gamma }$ = *d*γ/*d*t). As the strain rate increases, the viscosity decreases. In viscous materials, stress and strain are out-of-phase and a phase difference (δ) is π/2. A stress–strain relation of viscoelastic materials is in-between those of elastic and viscous materials. When materials are deformed, a phase difference between stress and strain is not either 0 or π/2. Measured stress of viscoelastic materials has in-phase and out-of-phase components in comparison with strain.

In the oscillatory measurement, a small magnitude of shear strain is first induced in an oscillatory mode in soft materials or fluids which are confined between two circular plates. Then, stress and strain of materials are examined simultaneously. By measuring the time lag of frequency Δ*t*, the phase angle shift δ is attained:

5 $$\updelta = \Delta t\omega$$

where ω is the frequency in radians per second (ω = 2π*ν*, ν is the frequency in Hz). A complex shear modulus G* of materials under oscillatory shear is written by;

6 $$G^* ( \omega ) = \tau ( t ) / \gamma ( t)$$

where τ(t) is shear stress. It is noted that G*(ω) is a function of the oscillation frequency ω. For viscous and viscoelastic systems, shear stress is in advance of strain by a phase difference of δ. Then, stress and strain have following forms;

7 $$\gamma (t)= \gamma_{\text{oi}} { \exp }\left( {i\omega t} \right)=\gamma_{\text{o}} { \sin }\omega t$$

8 $$\tau \, (t)\, \tau_{\text{oi}} { \exp }\left[ {i\left( {\omega t + \, \delta } \right)} \right]=\tau_{\text{o}} { \sin }\left( {\omega t + \delta } \right)$$

where γ_o_ and τ_o_ is the amplitude of imposed strain and measured stress, respectively. In a complete elastic system, the stress is exactly in phase with the strain (δ = 0), while in a complete viscous liquid, it is exactly out of phase with the strain (δ = 90°). For a viscoelastic system, the phase angle shift lies on a certain point between elastic and viscous systems. From the equations above, the following relations can be derived;

9 $$G^{\prime}= \left| {G^*} \right|{ \cos }\delta$$

10 $$G^{\prime\prime}= \left| {G^*} \right|{ \sin }\delta$$

11 $$G^* = G^\prime + iG^{\prime\prime}$$

**Fig. 2:**
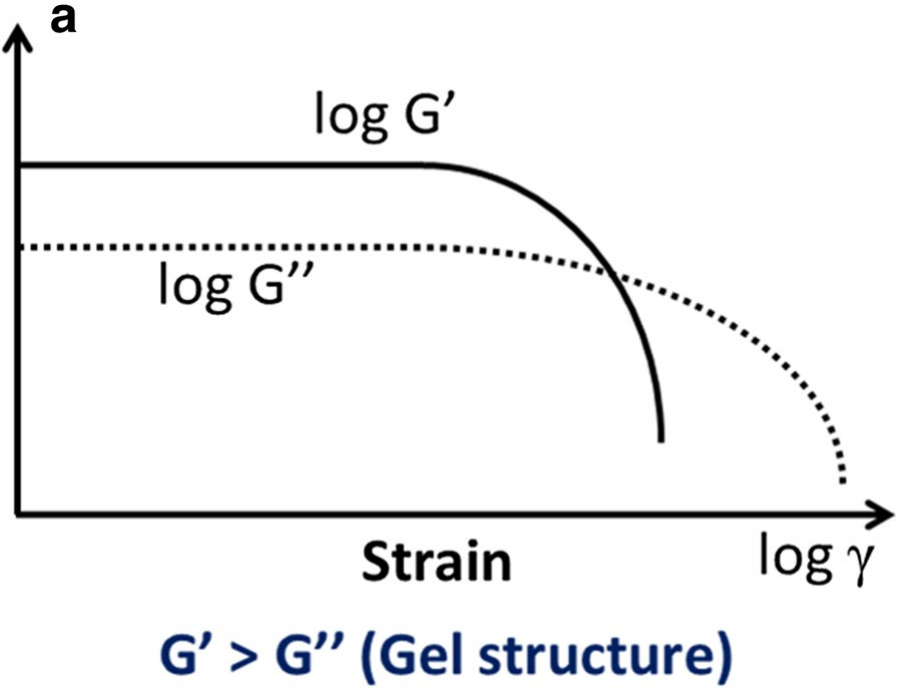
Changes in storage modulus (G′) and loss modulus (G″) as a function of strain in a typical viscoelastic system; this can be measured by a small amplitude oscillatory method

*G*′ and *G*″ are called storage modulus and loss modulus, which are measures of stored energy and dissipated energy during the cyclic deformation. Figure 2 shows a relative change in *G*′ and *G*″ as a function of strain in a typical viscoelastic fluid system. In a strain regime where *G*′ is larger than *G*″, colloidal suspensions behave like gel and particles have strong interactions to form a network structure. If *G*′ is smaller than *G*″, the interparticle interactions are weakened and colloidal suspensions exhibit a liquid-like behavior. Small amplitude oscillation experiment also provides a transition point from gel-like to liquid-like behavior of viscoelastic fluid system (*G*′ = *G*″).

## Experiment

### Materials synthesis

Na^+^-montmorillonite (Kunipia F, Kunimine Corp) was used as a starting clay material. It has the chemical formula Na_0.35_K_0.01_Ca_0.02_(Si_3.89_Al_0.11_)(Al_1.60_Mg_0.32_Fe_0.08_)–O_10_(OH)_2_·*n*H_2_O and a cation exchange capacity (CEC) of 100 mequiv/100 g. FeO(OH) (Aldrich), oleic acid (Mallinckro) and 1-octadecene were used to synthesize iron oxide nanoparticles. All the chemicals were used without further purification.

Oranopilization of the clay: the Na^+^-montmorillonite was dispersed in water containing the cationic surfactant, cetyl trimethylammonium bromide (CTAB) at room temperature. The weight ratio of the montmorillonite/water was 1:50 and the amount of CTAB was calculated to completely exchange cations in the montmorillonite. The temperature of the solution was then increased to 80 °C, and the solution was vigorously stirred for 4 h. The resulting CTAB intercalated clay was filtered and washed several times with distilled water before being dried at 60 °C in a vacuum for 24 h. Then the agglomerated particles were ground with a mortar.

Synthesis of the Fe_2_O_3_ nanoparticles [25]: Magnetite nanoparticles were synthesized in a three-neck flask equipped with a condenser, magnetic stirrer, thermocouple and heating mantle. A mixture of 0.178 g FeO(OH) fine powder (2.00 mmol), 2.26 g oleic acid (8.00 mmol) and 5.00 g 1-octadecene was heated to 320 °C and reacted for 1 h during vigorous stirring. The resulting oleic acid coated iron oxide nanoparticles were collected by centrifugation and were then washed several times with ethanol (EtOH). The residual liquid was frozen and sublimed at −50 °C in a vacuum for 24 h.

### Fluid preparation and characterization

Preparation of the magnetic fluid: The oleic acid coated magnetite nanoparticles were attached to the surface of organoclay plates in mineral oil. First, 0.5 g of the organoclay powder was slowly added to 20 ml mineral oil, and the mixture was sonicated to exfoliate organoclay. The resulting exfoliated organoclay plates were well dispersed in the mineral oil and 0.5 g oleic acid coated magnetite nanoparticles were added into the solution of exfoliated organoclay. The mixture was then sonicated for 1 h to promote the attachment of the magnetite nanoparticles to the surface of the organoclay.

The shape and crystal structure of the synthesized materials were characterized using x-ray powder diffraction (XRD, a Philips PW 1810 diffractometer with Cu Kα radiation, λ = 1.542 Å, 40 kV, 30 mA), transmission electron microscopy (TEM, Jeol 200CX), and photocorrelation spectroscopy (PCS) (Horiba LB 550). The magnetic properties of the materials were measured using a vibrating sample magnetometer (VSM, LakeShore 7400). Rheology measurements of the particle dispersed fluid were performed by a rheometer (Anton Parr, MCR 300) with a magnetorheological cell. To examine the effect of a magnetic field on the rheology of the fluids, a homogeneous magnetic field was set perpendicular to the direction of shear flow during the rheology measurement.

## Results and discussion

**Fig. 3:**
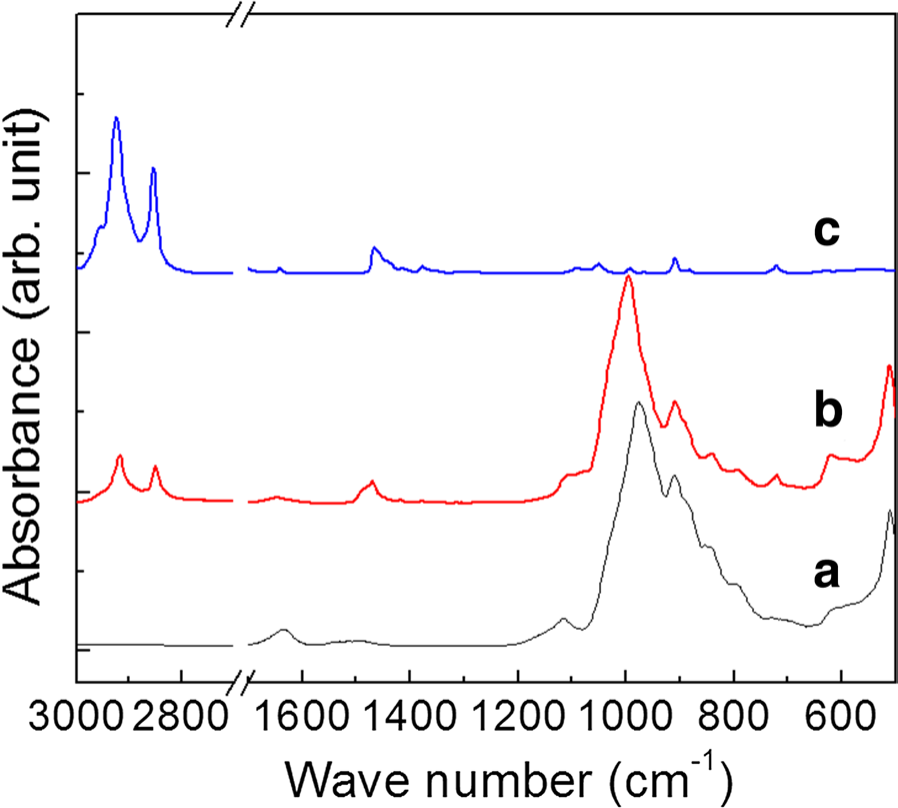
FT-IR spectrum of (*a*) pristine clay, (*b*) CTAB treated clay (organo clay), (*c*) OA-treated iron oxide nanoparticles; FT-IR measurement was conducted in ATR mode

Chemical moieties on the surface of particles are characterized using Fourier transform infrared spectroscopy (FT-IR). Figure 3 shows FTIR spectrum of (a) pristine clay, (b) CTAB treated clay (organoclay), (c) OA-treated iron oxide nanoparticles. All FT-IR measurements are conducted in ATR mode to collect signals mainly from the surface of chemically modified particles. In pure clay, a band between 800 and 1100 cm^−2^ is attributed to Si–O bond of a silicate layer. A small peak at 1627 cm^−1^ is due to the bending of OH that is attached to the clay surface. After CTAB is coated, O–H bending mode almost disappears and new peaks are found at 1470, 2850 and 2920 cm^−1^. They are related to bending and stretching of C-H bonds of a hydrophobic alky chain. Oleic acid coated iron oxide nanoparticles show IR responsive modes at 2854 and 2925 cm^−1^ which are assigned to the stretching vibration of C–H bonds of oleic acid. A change in FT-IR spectra shows that alkyl group and oleic acid are coated on the surface of clay and iron oxide nanoparticles and particles turn to be hydrophobic.

**Fig. 4:**
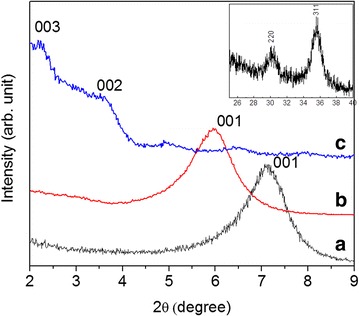
XRD patterns of (*a*) pristine clay, (*b*) CTAB-treated clay (organoclay) and (*c*) CTAB-treated clay which are decorated with OA-treated iron oxide nanoparticles. The inset shows XRD pattern in a 2θ range of 30–40° from (*c*)

**Fig. 5:**
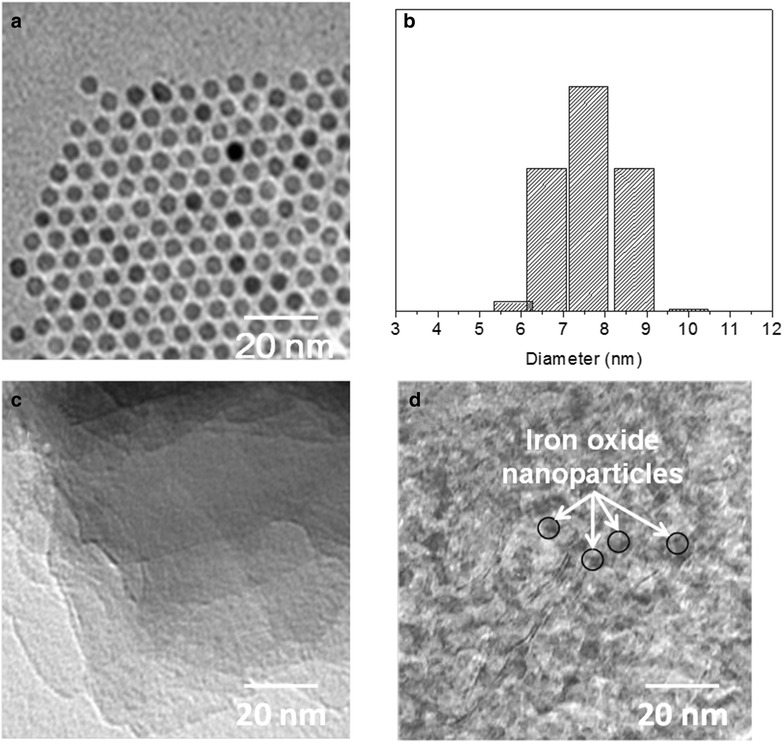
**a** TEM micrograph of OA-treated iron oxide nanoparticle, **b** a size distribution of nanoparticles (measured from Fig. 5a), **c** TEM micrograph of exfoliated clay and **d** TEM micrograph of OA-treated iron oxide and CTAB-treated clay

Figure 4 shows the x-ray diffraction (XRD) patterns for pristine clay, clay intercalated with CTAB (called organoclay) and composite particles that are organoclay decorated with OA-treated iron oxide nanoparticles. When the CTAB molecules are dissociated in water, cetyl trimethylammonium cations replace alkali or alkali earth cations staying the interlayer space of montmorillonite [26]. In Fig. 5, (*001*) reflection of the montmorillonite phase shifts from 2θ = 7.2° to 2θ = 5.9°. This indicates that a basal spacing normal to a basal plane of clay increases from 12.4 Å (pristine clay) to 14.8 Å (CTAB treated organoclay). An inset of Fig. 5 shows that a crystalline phase of iron oxide nanoparticles is magnetite. Presence of magnetite nanoparticles on CTAB treated clay also suggests that nanoparticles are well bonded to the organoclay through attractive interaction of hydrophobic surface groups. Since the basal spacing of organoclay (~1.5 nm) is much smaller than the diameter of magnetite nanoparticles (~7.5 nm), we speculate that nanoparticles mainly bonded to the surface of the exfoliated organoclay platelets.

Figure 5a show a TEM micrograph of OA-treated magnetite nanoparticles. Uniform spherical nanoparticles are found and their average size is smaller than 10 nm. A size of nanoparticles is measured from a TEM micrograph and its result is plotted in Fig. 5b. A mean diameter is 7.5 nm and most of nanoparticles have the diameter ranging from 6 to 9 nm. Figure 5c shows a TEM micrograph of montmorillonite after pristine clay is exfoliated. If clay is completely exfoliated, a thickness of a 2-dimensional layer can be smaller than 10 nm and an edge of the clay is transparent for an electron beam of TEM. The microstructure of the iron oxide nanoparticle decorated organoclay (i.e. clay coated with hydrocarbon moieties) is also examined using high resolution TEM (HR-TEM). Figure 5d shows that iron oxide nanoparticles with a diameter of about 7 nm are densely attached to the 2-dimensional surface of organoclay. Hence, even the edge of the composite particles is not transparent for the electron beam and complex features are found in TEM micrograph.

**Fig. 6:**
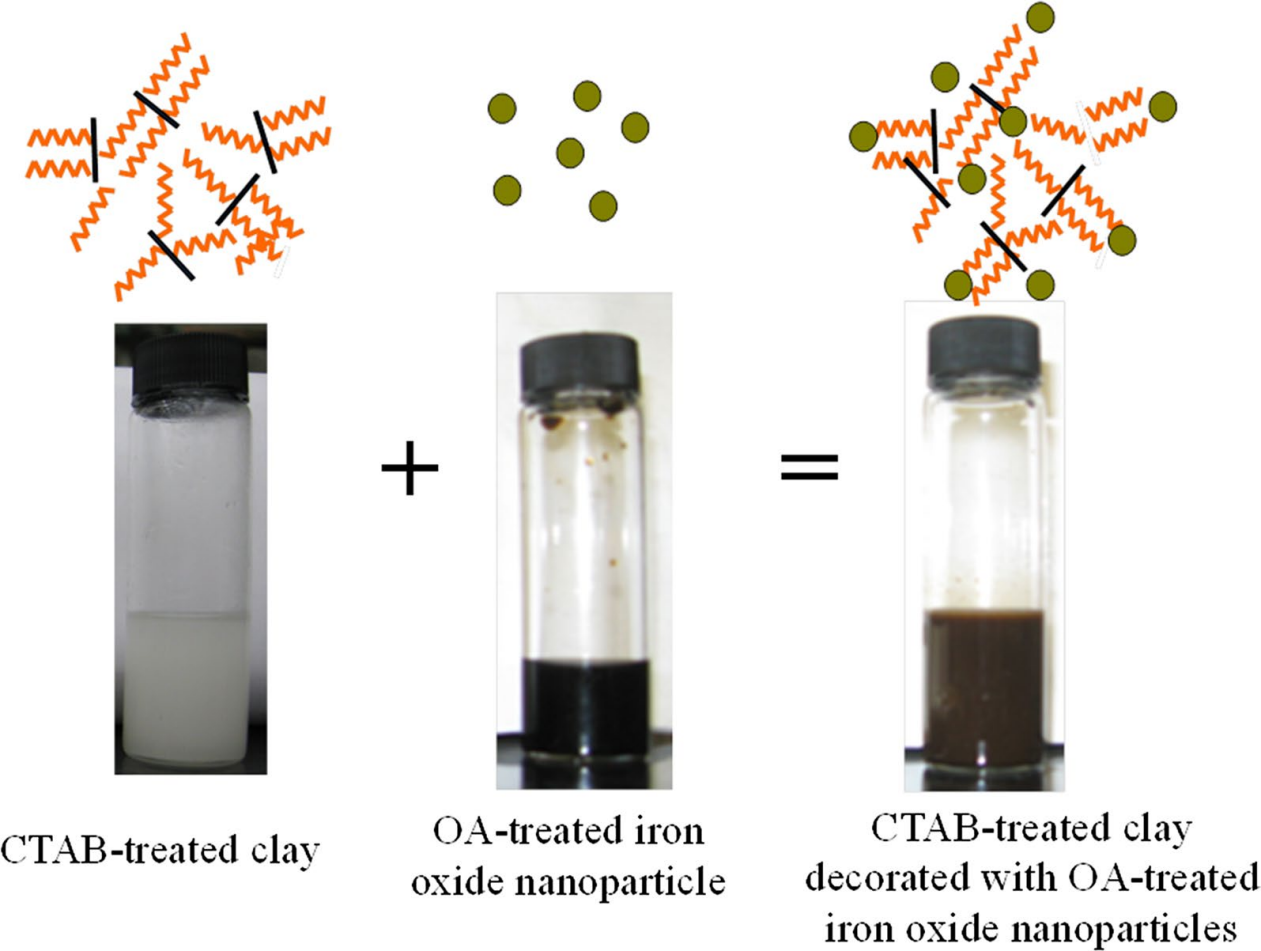
*Top* A schematic of CTAB-treated clay, OA-treated iron oxide nanoparticle and a composite of CTAB-treated clay and OA-treated iron oxide nanoparticle; *bottom* photographs of fluids consisting of mineral oil and different particles

Based on characterization above, surfaces of synthesized particles (CTAB treated organoclay, OA treated magnetite nanoparticles, and magnetite nanoparticle decorated organoclay) are illustrated in the top row of Fig. 6. Photographs of particle suspensions in an oil medium are also shown in the bottom row of Fig. 6. All particles are well dispersed in the mineral oil and segregation of particles in suspensions is not observed for 30 days.

**Fig. 7:**
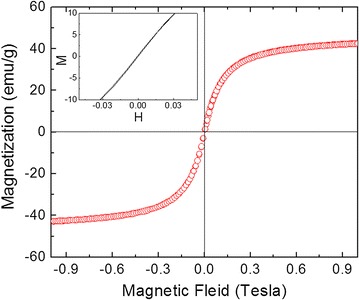
Magnetization (M) versus magnetic field (H) of composite particles consisting of OA-treated iron-oxide nanoparticles and CTAB-treated clay

Figure 7 shows the M–H curves of magnetite nanoparticle decorated organoclay. Saturation magnetization (σ_s_) of composite particles is 42 emu g^−1^, which is half the saturation magnetization of magnetite (~80 emu g^−1^) [27, 28]. This indicates that the magnetite content in the composite is about 50 wt%. Coercive field of the composite material in an inset of Fig. 7 is almost zero, which agrees well with a well-known very small coercive field of the magnetite nanoparticles [29, 30]. These small coercive field and remnant magnetization are due to the size of magnetic nanoparticles that exhibit superparamagnetism. When the particle size becomes smaller than 10 nm, thermal agitation at room temperature overwhelms the magnetic interaction between nanoparticles at low magnetic field. If the size of magnetic particles is in a micrometer regime, the coercive field and remnant magnetization are not close to zero at room temperature. In large particles, therefore, the tunability of magnetization is decreased and the irreversibility of M–H curve is increased. This may be harmful in precisely controlling the rheological behavior of the oil containing magnetite nanoparticle–organoclay mixture by applying magnetic field.

**Fig. 8:**
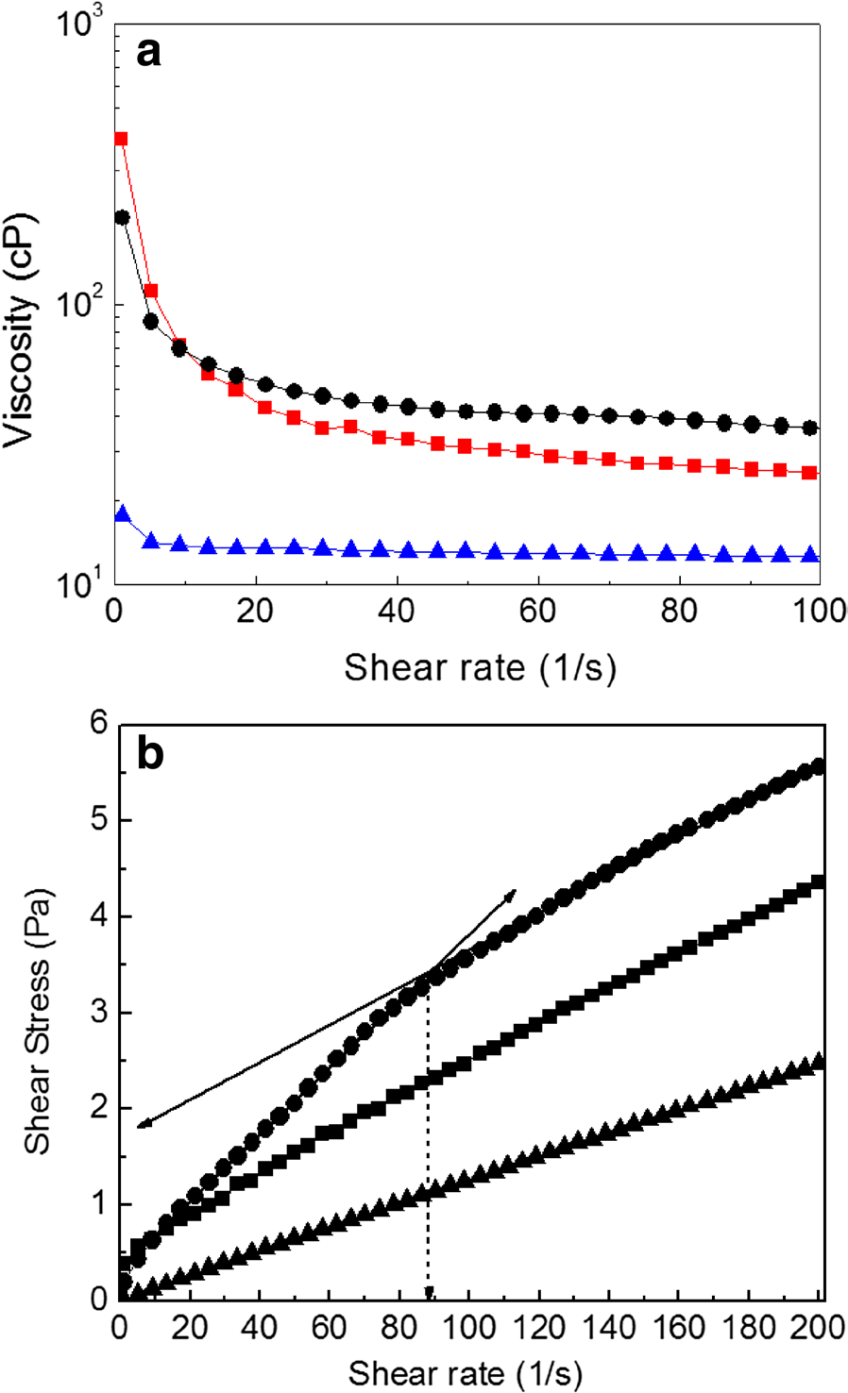
**a** Viscosity of fluids measured and **b** shear stress versus shear rate of fluids from an oscillatory measurement; (*filled circle*) fluid of OA-treated iron-oxide and CTAB-treated clay, (*filled square*) fluid of CTAB-treated clay and (*filled triangle*) fluid of OA-treated iron oxide nanoparticles

The rheological properties of the mineral oil based fluids containing 1 wt% OA treated magnetite nanoparticles, CTAB treated organoclay, and magnetite nanoparticle–organoclay mixture were also measured. Figure 8 shows a change in viscosity and shear stress as a function of shear rate. Both the CTAB treated organoclay fluid and iron oxide nanoparticle–organoclay mixture fluid show thixotropic behavior. Specifically, the viscosity of fluids dramatically decreased as shear rate increases. A dramatic decrease of the viscosity in a high shear rate region indicates that the organoclay and nanoparticle decorated organoclay are interconnected to form a network structure in a static state. In contrast, the fluid containing 1 wt% OA-treated iron oxide nanoparticle exhibited typical Newtonian fluid behavior, where viscosity is independent of shear rate. When the shear rate is 80 1 s^−1^, the room-temperature viscosity is 12 centipoise (cp) for the fluid of 1 wt% OA-treated iron oxide nanoparticle, 26 cp for the fluid of 1 wt% CTAB-treated clay and 38 cp for the fluid of 1 wt% nanoparticle decorated organoclay. Network formation of particles at a zero shear rate and breakdown of the network at a high shear rate region are more quantitatively characterized by measuring shear stress versus shear rate relation. Shear stress curves in Fig. 8b consists of two different regimes. When the shear rate was smaller than 85 1 s^−1^, the slope of the shear stress–shear rate curves was larger for the nanoparticle decorated organoclay fluid and the bare organoclay fluid than for the magnetite nanoparticle fluid. The higher stress needed to produce the shear strain in the organoclay based fluids can be attributed to the unique network formation capability of the organoclay in the fluid. As the shear rate becomes larger than 85 1 s^−1^, the slope of the stress–strain curve becomes similar in all samples. Since the organoclay cannot be interconnected in a high shear rate regime, the network structure of the clay is broken and a difference in the rheological properties among three fluids becomes negligible.

**Fig. 9:**
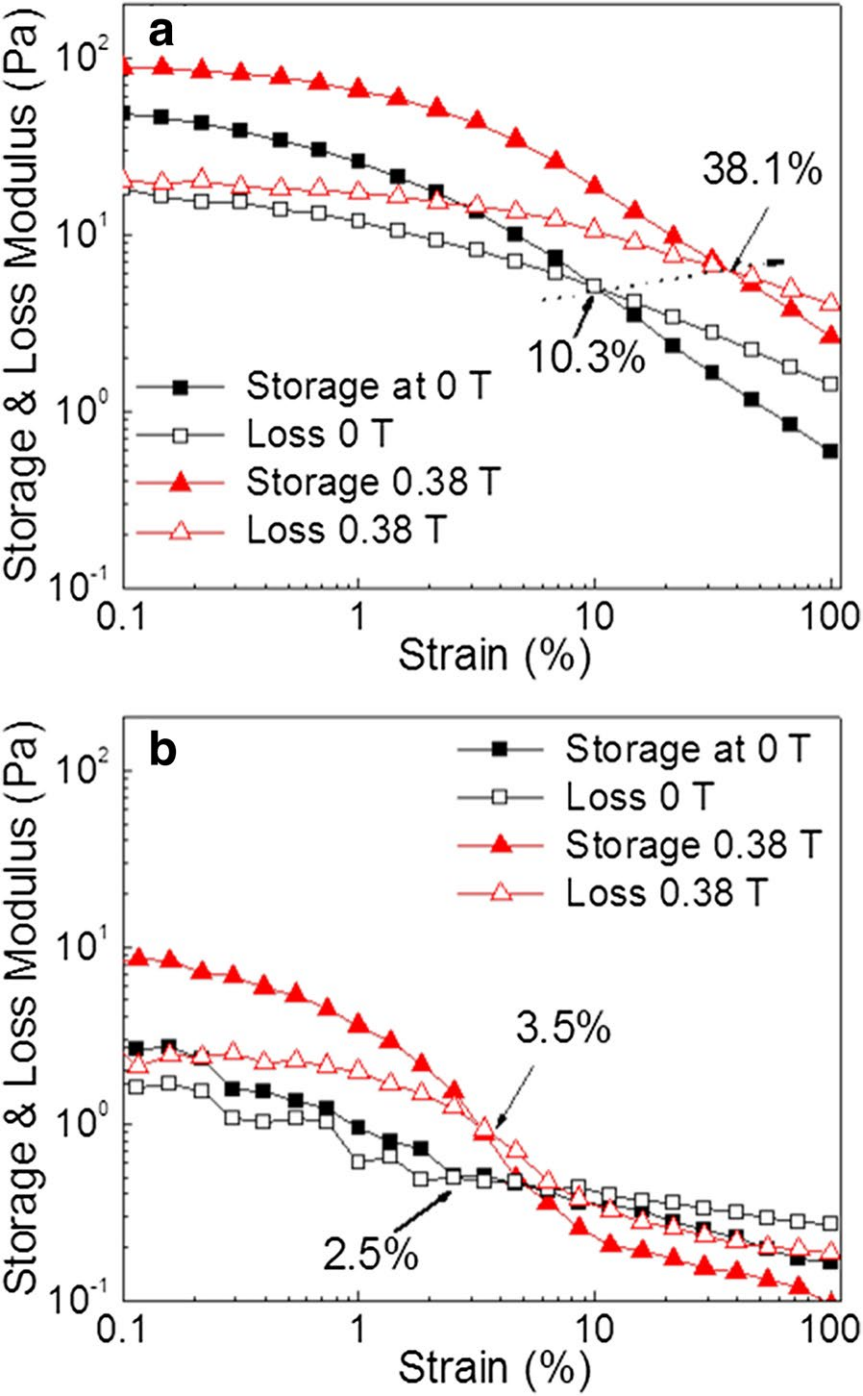
**a** Storage modulus and loss modulus versus strain of the fluids containing CTAB-treated clay coated with OA-treated magnetite nanoparticles and, **b** storage modulus and loss modulus versus strain of the fluids containing OA-treated magnetite nanoparticles; the oscillatory measurement was conducted with and without magnetic field 0.38 T

Since the magnetic particles are strongly bonded to the clay using a chemical interaction, the clay moves with the magnetic nanoparticles when they are exposed to a magnetic field. Therefore, decorating the surface of the organoclay with magnetite nanoparticles provides the freedom to tune the rheological properties of the fluid by applying a magnetic field. In this study, the magnetorheological properties of the fluid were measured while the fluid was in an oscillating motion. This oscillatory technique is widely used in characterizing the viscoelastic properties of fluid systems [29, 31–33]. An oscillation frequency is fixed at *ω* = 5 rad s^−1^, and a sweeping strain ranged from 0.01 to 100%. During the oscillatory measurement, a magnetic field of 0.38 T is applied to study the effect of a strong bond between magnetite nanoparticles and clay on a magnetorheological behavior. A fluid containing only OA-treated magnetite nanoparticles is also characterized as a reference. Figure 9 shows that magnetic field increases the storage modulus of the nanoparticle decorated organoclay fluid by three times. In addition, a linear viscoelastic (LVE) behavior is observed in the magnetic field applied fluid when the sweeping strain was small. Storage modulus of the viscoelastic fluid is related to the elastic internal motion of a solid component [33]. Nearly constant storage modulus in a small strain regime shows that the magnetic field induces a stable network of the solid components and the fluid behaves like solid. As a shear strain increases, the elastic behavior of the solid network is not maintained, even under a magnetic field, and the loss modulus increases. A critical point at which the storage modulus is same as the loss modulus is called the “flow point” [31]. When a magnetic field of 0.38 T is applied, the flow point of the fluid containing the nanoparticle decorated organoclay moved from 10.3 to 38.1%. In contrast, the fluid containing only OA treated magnetite nanoparticles does not exhibit a change in the critical strain. Without clay, the flow point is observed at about ~3% with or without magnetic field. An increase in the strain of flow point in composite particle fluids attests that the nanoparticle decorated organoclay forms a strong network structure under magnetic field and the viscoelastic properties of the fluid can be controlled by changing magnetic field strength.

## Conclusion

We examined the viscoelastic behavior of a stable non-aqueous fluid containing where clay base composite particles are dispersed. The composite particles are CTAB-treated organoclay which is decorated with OA-treated iron oxide nanoparticles. CTAB treatment and OA treatment are performed to make surfaces of clay and iron oxide nanoparticles hydrophobic. Due to hydrophobic attraction, OA-treated nanoparticles are well attached to the surface of montmorillonite. Magnetite nanoparticle decorated organoclay is well dispersed in oil media and the fluid of composite particles exhibits a viscoelastic behavior. When external magnetic field is applied, superparamagnetic properties of magnetite nanoparticles causes realignment of composite clay particles in the fluid and a transition to a liquid-like observed is observed at higher shear strain. Our results suggest that the viscoelastic property of the composite particle fluid is tunable and an elastic contribution is increased by increasing magnetic field strength.
